# The after-school sedentary behavior status among children and adolescents with intellectual disabilities

**DOI:** 10.3389/fpsyt.2022.1049180

**Published:** 2022-12-01

**Authors:** Yaqing Yuan, Jianing Ding, Chao Wang, Shaohua Zhang, Yinping Wang, Yang Liu, Jingmin Liu

**Affiliations:** ^1^Division of Sports Science and Physical Education, Tsinghua University, Beijing, China; ^2^College of Sports and Health, Shandong Sport University, Jinan, Shandong, China; ^3^Department of Physical Education, Fujian Agriculture and Forestry University, Fuzhou, Fujian, China; ^4^Department of Health Management, Yongan Prison of Fujian Province, Yongan, Fujian, China; ^5^Department of Physical Education, China Disabled Persons’ Federation, Beijing, China; ^6^Beijing Tongzhou Nationalities Primary School, Beijing, China; ^7^Department of Physical Education, Shandong Jianzhu University, Jinan, Shandong, China

**Keywords:** after-school, sedentary behavior, intellectual disabilities, children and adolescents, health promotion (HP)

## Abstract

**Background:**

There is evidence that the after-school period plays an essential role in accumulating sedentary behavior (SB) among children and adolescents, as well as implementing potential interventions. However, relatively little is known regarding SB status of children and adolescents with intellectual disabilities (ID) during the after-school period. The purpose of this study was to investigate the total level and specific pattern of the after-school SB among children and adolescents with ID.

**Methods:**

The after-school SB status among 325 children and adolescents with ID was evaluated by the parent-reported Children’s Leisure Activities Study Survey-Chinese edition questionnaire.

**Results:**

Parents of children and adolescents with ID reported approximately 204 min/day of after-school SB. Specifically, the longest time of the after-school period was spent performing the screen-based SB (84 mins/d). This was followed by recreational SB and educational SB (50 and 30 mins/d, respectively). The children aged 6–12 years old engaged more time in recreational SB than adolescents aged 16–18 years old (*p* < 0.05) during the after-school period. Further, the data indicated that 37.5% of children and adolescents with ID achieved the guideline limitation of 2-h-maximum screen-based SB during the after-school hour.

**Conclusion:**

Children and adolescents with ID spent a large portion of the after-school period in SB, particularly engaged in more time on after-school screen-based SB. Future efforts should focus on developing and implementing period-specific interventions designed to reduce after-school SB in the segment of this population.

## Introduction

The review by Carson et al. showed that it was crucial and beneficial for children and adolescents to engage in moderate-to-vigorous physical activity (MVPA) for 60 min every day (1). However, research focusing on other health-related behavior for the remaining 23 h of the day has traditionally been scarce, although it is fast increasing. Sedentary behavior (SB) is one of the particular critical behaviors, which is defined as any waking behavior (e.g., in a seating, reclining, or lying down posture) with an energy expenditure ≤ 1.5 METs (metabolic equivalents) (2).

It has been demonstrated in numerous studies that SB is increasingly linked to all-cause mortality, overweight/obesity, type II diabetes, lower physical fitness, cardiovascular disease, and some cancers, independent of physical activity (PA) (3–5). Furthermore, the findings from Carson et al.’s review have also revealed that different types of SB (e.g., watching TV, using the computer, or doing homework) may have various impacts on health (1).

More recently, SB has been common among children and adolescents worldwide (6). Based on solid evidence from two national-level datasets in Canada and the United States, SB was found to account for a substantial proportion of waking times (50–60%) of the day in children and adolescents (7, 8). To reduce the adverse health impacts of SB, the World Health Organization (WHO) suggests that 5–17-year-old children and adolescents should limit screen-based SB to under 2 h per day (9). The 24-Hour Movement Guideline also recommends that children and adolescents minimize time spent in SB (10). These recommendations apply to most children and adolescents, including those peers with ID.

Prior studies have found that children and adolescents with ID spent more time sedentary than their counterparts without ID (11–13). Esposito et al. and Phillips et al. also pointed out that the high prevalences of obesity, poor fitness, and functional limitations experienced by children and adolescents with ID, possibly be related to their high levels of SB (11, 12). A high level of SB may, in turn, lead to a further increase in adverse health outcomes among children and adolescents with ID. Under such circumstances, children and adolescents with ID may not be able to avoid the negative health effect of long-term SB engagement, even though they could follow the MVPA recommendations. In addition, it is essential to note that in the general population, SB appears to increase from childhood to adulthood (6, 8). The existing data indicate that, from the life-cycle perspective, individuals with ID often experience premature aging issues (14). These early onset aging may affect the pattern of SB development throughout the lifespan of children and adolescents with ID. Thus, there is a great need for a more comprehensive and detailed look at the characteristics of SB among children and adolescents with ID.

A range of research has revealed that the specific time of the day that can potentially make a significant contribution to children and adolescents’ daily SB is the after-school period (15, 16). Children and adolescents are often not bound by school schedules during this period. They have more choices about their behavior than during the school day. However, to our knowledge, there was only one study that focused on the field of after-school SB among children with ID (17). Foley and McCubbin found that 7–12 year-old children with ID spent time on watching TV or on the computer was no different than their peers without ID, but it was encouraging that the majority of the children did not exceed the limitation of 2 h/d. It is worth noting that the study was published more than 10 years ago, and given the small sample size, it is uncertain whether its findings are still valid in terms of reflecting the after-school SB of children with ID today. In this context, our study was conducted to understand the overall level SB and detailed information on different types of SB during the after-school period among children and adolescents with ID aged 6–18 years through a relatively large sample sampling, as a step toward establishing and collecting baseline data to provide targeted period-specific strategies for health promotion in this population.

## Materials and methods

### Study design

A cross-sectional descriptive design assessed the after-school SB status among children and adolescents with ID by using parent-reported surveys. The survey was completed between September 13 and December 24, 2021. All parents involved in this study, as well as their children, were explicitly advised that participation was entirely voluntary. All the data were centrally analyzed anonymously. The study was conducted in accordance with the Declaration of Helsinki Principles, and approved by the ethics committee of the university.

### Participants

A convenience sampling strategy focusing on the parents of children and adolescents with ID was utilized among special education schools in Shandong Province. The following were inclusion criteria for the parents: (1) their child came from day school; (2) their child was between the ages of 6 and 18; (3) their child could walk without any help; and (4) their child did not experience coexisting cerebral palsy, autism, and other sensory impairments. The ID level was categorized as profound [intelligence quotient (IQ) < 25], severe (IQ of 25–39), moderate (IQ of 40–54), and mild (IQ of 55–70) (18). Additionally, children and adolescents with ID were divided into three age subgroups, 6–12, 13–15, and 16–18 years old, which matched the Chinese school education system’s definition of the age range for the primary, junior middle, and junior high schools (19).

### Procedures

Based on the most recent data, 33 special education schools primarily recruit children and adolescents with ID in Shandong Province of China (20). The school-keepers from 16 special education schools were contacted through the help of the China Disabled Persons’ Federation. Finally, 10 special education schools accepted the invitation and consented to participate in the study. Invitations containing the purpose and content of this study were sent out by headmasters to the legal guardians of children and adolescents with ID. In the meantime, the objective of the present study was explained briefly to children and adolescents with ID. Verbal permission was also obtained in their schools prior to data collection. Once the caregivers gave written informed consent, the teachers contacted them to come to school to complete the questionnaire. The parents were given step-by-step instructions by trained teachers and researchers through the parents’ meeting on how to complete the questionnaire. They were also given plenty of time for questions.

### Sedentary behavior assessment

After-school SB of children and adolescents with ID was assessed using reliable items from the Children’s Leisure Activities Study Survey-Chinese edition (CLASS-C) questionnaire, which is widely used in China and has good reliability and validity (21). Li et al. also stressed that no statistically significant difference was observed in measurement between the CLASS-C questionnaire and accelerometry (21). In this study, prior to data collection, a pretest CLASS-C questionnaire was completed by a sample of 30 parents from one special education school in the Jinan City of Shandong Province. The pretest showed that the CLASS-C questionnaire possessed sound reliability (Cronbach’s *a* = 0.752). The CLASS-C questionnaire contained a list of twelve common after-school SB in addition to demographic information that consisted of gender, age, height, weight, and ID level. Based on the manifestation of behavior, these common after-school SB can be classified into four categories: screen-based SB (watch TV, play video/computer games, and surf the Internet), educational SB (do homework and read books), recreational SB (play with toys, listen to music, play musical instruments, play card games, and art activities) and social SB (chat while sitting stationary and make phone calls). In addition, as this study only focused on the after-school period, parents were requested to provide the amount of time their child spent on the specific after-school SB pattern from Monday to Friday (five consecutive days). After-school hours, in this study, were defined as the period between the end of school bell time and bedtime.

The parent-reported survey was chosen in the current study for the following three reasons. First, children and adolescents with ID may not completely understand the content of the questionnaire, and hard to finish the questionnaire independently. In documenting their child’s activities, Burdette et al. found that parents were able to give accurate estimates (22). Second, the questionnaire measurement could gather information about the type of SB–a capability not provided by device-based assessments, such as accelerometers. Further, large-scale epidemiological studies often consider questionnaires as a cost-effective alternative to accelerometers.

### Statistical analyses

All data analyses were conducted on SPSS version 25.0 for Mac (SPSS Inc., Chicago, IL, USA). Descriptive statistics were calculated for all study variables (gender, age, weight status, and ID level). All variables were examined whether they were normally distributed by means of the Kolmogorov–Smirnov test. Due to the non-normal distribution of SB variables, the Mann–Whitney *U* test was utilized to compare the SB differences between gender and weight status. The Kruskal–Wallis test was applied to analyze differences in SB between age and ID level. For prevalence, estimates were calculated as the percentage of children and adolescents with ID who achieved the 2-h-maximum screen-based SB time limitation in the guideline. Differences in the prevalence of screen-based SB time limitation by gender, age, weight status, and ID level were tested using a binary logistic regression model. Prevalence estimates, odds ratio (OR), and corresponding 95% confidence interval (CI) were calculated from the logistic regression. *P*-values < 0.05 were considered statistically significant for all analyses.

## Results

### Demographic analysis

Data in relation to after-school SB from 325 children and adolescents with ID were provided from parents’ reports. A total of 220 (67.7%) children and adolescents with ID were boys, aged 12.4 ± 3.3, and 105 (32.3%) were girls, aged 12.7 ± 3.8. In addition, 104 (32.0%) of them were moderate ID, 167 (51.4%) were severe ID, and 54 (16.6%) were profound ID.

### After-school sedentary behavior level among children and adolescents with intellectual disabilities

The daily time of total after-school SB and specific types of after-school SB among children and adolescents with ID are presented in Table 1. Children and adolescents with ID engaged in approximately 204 min of total after-school SB per day. In detail, they spent approximately 84 mins/d during the after-school period in the screen-based SB. Furthermore, after-school children and adolescents with ID performed about 50 mins/d of recreational SB and 30 mins/d of educational SB, respectively. Additionally, no significant differences were observed in total after-school SB or particular after-school SB for children and adolescents with ID by gender, weight status, and level of ID (*p* > 0.05). An exception, however, was seen during the after-school period where the youngest-aged (6–12 years old) group engaged more time in recreational SB than the oldest-aged (16–18 years old) group (*p* < 0.05).

**TABLE 1:** Daily minutes of total after-school SB and specific types of after-school SB among children and adolescents with ID [M (P25–P75), min/days].

	Total SB	Educational SB	Screen-based SB	Recreational SB	Social SB
Both gender	204 (96–252)	30 (0–60)	84 (48–120)	50 (0–60)	0 (0–18)
Gender					
Boys	216 (117–252)	45 (0–60)	84 (48–120)	50 (0–60)	0 (0–17)
Girls	195 (84–252)	27 (0–68)	60 (48–120)	48 (6–60)	0 (0–24)
Age					
6–12	228 (98–278)	36 (0–60)	84 (36–120)	50 (12–84)	0 (0–24)
13–15	195 (108–240)	30 (0–60)	96 (50–120)	50 (4–60)	0 (0–10)
16–18	180 (96–240)	30 (0–84)	60 (60–120)	12 (0–60)	0 (0–48)
ID level					
Moderate	228 (120–254)	46 (0–72)	72 (60–130)	50 (5–84)	0 (0–12)
Severe	195 (110–246)	30 (0–60)	90 (48–120)	50 (6–60)	6 (0–24)
Profound	157 (50–264)	18 (0–72)	66 (21–120)	24 (0–60)	0 (0–30)
Weight status					
Normal weight	192 (96–264)	30 (0–60)	72 (36–120)	50 (12–60)	2 (0–24)
Overweight/Obesity	216 (105–245)	45 (0–60)	120 (60–120)	36 (0–72)	0 (0–17)

Table 2 displays the detailed prevalence estimates of screen-based SB during the after-school period by gender, age, weight status, and ID level of children and adolescents with ID. Overall, 37.5% of them achieved the 2-h-maximum screen-based SB time limited in the guideline during the after-school time. No statistically significant differences were found in the prevalence estimates of screen-based SB by gender, age, weight status, and ID level during the after-school period from the logistic regression model results (Table 3).

**TABLE 2:** Prevalence estimates of after-school screen-based SB time limitation among children and adolescents with ID by gender, age, weight status, and ID level.

	Achieving the screen-based SB time limitation (95% CI)
Both gender	37.5 (32.2–42.8)
Boys	39.1 (32.6–45.6)
Girls	34.3 (25.1–43.5)
Age	
6–12	35.2 (27.3–43.2)
13–15	39.7 (30.6–48.7)
16–18	38.8 (26.8–50.8)
Weight status	
Normal weight	37.8 (30.6–44.9)
Overweight/Obesity	37.2 (29.3–45.2)
ID level	
Moderate	37.5 (28.0–47.0)
Severe	38.9 (31.5–46.4)
Profound	33.3 (20.3–46.3)

**TABLE 3:** Differences in prevalence of achieving the after-school screen-based SB time limitation among children and adolescents with ID by gender, age, weight status, and ID level.

	Achieving the screen-based SB time limitation OR (95% CI)
Both gender	
Girls	Referent
Boys	1.225 (0.747–2.011)
Age	
6–12	Referent
13–15	1.234 (0.734–2.074)
16–18	1.242 (0.674–2.291)
Weight status	
Normal weight	Referent
Overweight/Obese	0.951 (0.598–1.510)
ID level	
Moderate	Referent
Severe	1.066 (0.641–1.773)
Profound	0.820 (0.403–1.669)

## Discussion

This study described the overall level and specific pattern of the after-school SB in children and adolescents with ID, derived from parent-reported data. Based on the daily routine of school days, we deduce that children and adolescents with ID leave school at 4:00 p.m. and sleep at 10:00 p.m. After removing approximately 1 h of time needed to get home and have dinner, there are approximately 5 h left during this period. The results of this study indicated that during the after-school time, present children and adolescents with ID had 204 mins/d of SB, which was equivalent to 3.4 h every day. Excessive after-school SB is a serious problem for children and adolescents with ID. It was evident that they spent approximately 70% of their after-school time in SB, which was relatively higher than the findings of Arundell et al.’s systematic review focused on after-school SB among typically developing (TD) children and adolescents (15). Arundell et al.’s study revealed that TD children spent between 41 and 51% of the after-school period sedentary, and TD adolescents engaged 57% of the after-school time in SB (15). The earlier reports of poor health conditions associated with SB in children and adolescents with ID may explain part of the higher level of their after-school SB (11, 12). However, it is hard to confidently conclude whether the after-school SB of children and adolescents with ID differs from TD peers due to the fact that difference in the after-school period definitions and the measurement tools between the present study and the systematic review study. Additionally, existing data focusing on the overall SB level appear conflicting in the literature. Foley et al., Whitt–Glover et al., and Pitchford et al. concluded that no significant difference was found in the overall SB between children and adolescents with ID and TD peers (17, 23, 24). In contrast, a study from Poland based on a large sample showed that children and adolescents with ID had significantly longer SB than their counterparts (25). The reasons for this may be related to the choice of SB measurement tools, the size and type of sample with ID, and the use of SB cut-off points between studies. Therefore, caution is needed when making comparisons across different studies.

A key finding of this study was that screen-based SB was the most common form of after-school SB for children and adolescents with ID. The results found that the amount of time spent on screen-based SB after school was approximately 84 mins/day, which made up 41.2% of the total after-school SB level. This finding is similar to that reported in a previous study by Foley et al. In Foley et al.’s study, children with ID spent the majority of their after-school time watching television and computers for a combined 82 ± 64 mins/d (17). The findings of Adelantado–Renau et al. (26) highlighted that screen-based SB was the most prevalent behavior for TD children and adolescents in their daily lives. According to the review study by Carson et al., prolonged screen-based SB time was linked with adverse health effects (1). In detail, higher screen time/frequency was associated with unfavorable body composition, lower fitness performance, lower self-esteem, and higher risk of clustered cardiometabolism (1). A gradient was also observed across the different health indicators, showing that less SB, particularly screen time, was related to better health (1). In addition to the screen-based SB, the present study found that the recreational and educational SB comprise 39.2% of the total after-school SB level. Of note, it is possible several of these particular SB occur concurrently during the after-school period. For example, some learning and leisure activities are screen-based devices, such as reading books on pads, doing homework on the computer, listening to music on phones, and drawing pictures on the laptop. Therefore, screen-based devices usage should be limited, except for essential learning or leisure activities during the after-school period. It is also recommended to choose non-screen learning and entertainment activities instead of screen-based learning and entertainment activities. Moreover, from the point of view of the type of after-school SB, although the time accumulation and energy expenditure of SB are similar between educational SB and recreational SB, there may be specificity in the determinants and biological effects of health consequences of positive and negative SB. It is therefore challenging to effectively distinguish between different particular SB pattern and their health benefits in future studies.

In the present study, one unanticipated finding was that children and adolescents with ID in the youngest-age group engaged in more recreational SB compared to those in the oldest-age group during the after-school period, which indicated that there might be different after-school SB patterns among children and adolescents with different age ranges. This result, therefore, needs to be interpreted with some caution because the sample size across age groups in this study was uneven, as well as investigating SB status only during the after-school period. In addition, it is clear that the evidence from the present stage of the study does not establish whether age is an influential factor in the SB of children and adolescents with ID. Esposito et al. highlighted that the time of SB per week for adolescents with ID usually increased with age (11). One study from Japan also noted that children with ID aged 11–12 years old engaged significantly more time in SB than those aged 7–8 years old (27). However, Foerste et al.’s study outlined that there was no correlation between SB and age in adolescents with ID (28). It is worthy to mention that the above-mentioned studies recruited samples with Down’s syndrome (DS). Phillips and Holland found that individuals with DS are significantly more sedentary than those with ID without DS. Furthermore, it has been noted that individuals with DS engaged in lower PA and fitness levels, leading to cardiac chronotropic incompetence, impaired autonomic function, low muscular strength, and muscle hypotonia compared to individuals with ID without DS (12). Therefore, more research is needed to provide an in-depth analysis of the relationship between age factors and SB level/specific SB pattern at different periods of the day (e.g., at school or after school) based on a clear sampling (e.g., recruitment of samples with ID without DS or samples with ID only) type.

Several theories have been applied to facilitate the investigation of behaviors and their correlates. The ecological model theory suggests that behavior is influenced by intrapersonal factors as well as social/cultural and physical/policy environment (29). In this study, personal, family, and environmental factors could contribute to excessive after-school SB among children and adolescents with ID. As far as personal factors are concerned, on the one hand, children and adolescents with ID may have skeletal development and motor development issues that limit their activities (30). On the other hand, because of their cognitive deficits, they have difficulty recognizing the adverse health effects of SB and planning/organizing their after-school activities. Furthermore, a lack of interest in sports activities and social difficulties may also be factors contributing to higher levels of after-school SB among children and adolescents with ID. Regarding family factors, Izquierdo–Gomez et al.’s pointed out that the mother’s education, work status, and socio-economic status were associated with total SB time and watching TV time among adolescents with DS (31). Additionally, overprotective or worried parents could restrict their children’s range of activities or deny them the opportunity to participate in sports activities, making them more anxious and vulnerable (30). Therefore, parents’ understanding of the harmful effects of SB and their behavioral habits may influence their children’s behavior. To reduce the after-school SB of children and adolescents with ID, parents’ awareness of the value of after-school sports activities should be raised, their role in the healthy development of their children should be clearly defined, and their role as role models should be fully explored. The environment in the community and the facilities at home can also impact the after-school SB of children and adolescents with ID. According to Izquierdo–Gomez et al.’s study, total SB levels were positively correlated with the number of bedrooms, the presence of a garden, and a walkable neighborhood (31), which give direction to the identification of factors relevant for family or community-based interventions. Hence, in summary, children and adolescents with ID may experience high levels of after-school SB because of a combination of individual, family, and environmental factors.

One situation that cannot be ignored is that children and adolescents with ID spend half of their waking hours at school on weekdays. Traditionally, the classroom environment has been related to children and adolescents with ID spending long periods of time sitting. Consequently, the after-school period represents a vital part of the day for them. In general, they are not restricted by school schedules after school, and they may have some choices between active and sedentary options during these discretionary periods of the day. Furthermore, after-school SB may also contribute to daily SB level and affect the health of children and adolescents with ID. Hence, the targeted period-specific interventions given may be effective. For example, outdoor plays may provide a feasible opportunity for children and adolescents with ID to take some exercise or participate in sports activities and subsequently reduce SB during the after-school period. Also, Robinson et al. offered after-school dance classes to TD children, which had been shown to successfully reduce their screen-based SB (32). These directed at after-school interventions may be adapted to children and adolescents with ID and have the potential to change their after-school SB status. Further, two previous studies took different approaches to intervene on SB status in children and adolescents with DS or ID at the community level and school level, respectively. Ulrich et al. conducted a 7-week bicycle intervention for children and adolescents with DS in the community setting, with the results indicating that children and adolescents with DS who learned to ride engaged significantly less time in SB compared to their peers in the control group (33). A study from Hong Kong, China examined the effectiveness of active video games intervention strategy on PA level, motor proficiency, and body composition. The result of the study showed that, compared to the control group, children with ID in the intervention group had a decrease in SB after a 12-week intervention (34). However, the long-term effectiveness of these interventions in reducing SB and whether they are also applicable to the period after school need to be further investigated.

There are some limitations to this study. First, data were collected from special education schools in the fall semester. Previous studies showed that children were found to be more sedentary in the winter than in the spring or summer (35, 36). Therefore, this surveillance data may only represent after-school SB at a specific period of the year and may not be generalizable throughout the entire school year due to the after-school SB may be associated with the seasonal variation. Second, the convenience sample used from northern China may not be representative of the entire children and adolescents with ID in China. Additionally, the unbalanced sample size of each ID severity may have some influence on the interpretation of the results of the present study. Thus, a more complete sampling strategy is necessary for future research. Third, due to the cross-sectional study’s design, no causal relationship can be established. Therefore, in order to clarify and understand the trend and patterns of change in after-school SB over time, this requires future prospective analysis in further large longitudinal studies focusing on investigating the prevalences, trajectories, and determining factors of the after-school SB. Finally, notwithstanding these limitations, this investigation provides invaluable information to understand the pattern and distribution of after-school SB among children and adolescents with ID.

## Conclusion

Overall, the findings of this study highlighted that children and adolescents with ID spent a high level of SB during the after-school period. Among varieties of types of after-school SB, children and adolescents with ID particularly engaged in more time on after-school screen-based SB. A certain number of them exceeded the 2-h-maximum limit for screen-based SB time during the after-school period. Thus, it is necessary to implement period-specific strategies to reduce SB among the vulnerable population.

## Data availability statement

The data are not publicly available due to privacy or ethical restrictions. Requests to access these datasets should be directed to YY, crystal_267@163.com.

## Ethics statement

The studies involving human participants were reviewed and approved by the Ethical Committee of Shandong Sport University, China. Written informed consent to participate in this study was provided by the participants’ legal guardian/next of kin. Written informed consent was obtained from the minor(s)’ legal guardian/next of kin for the publication of any potentially identifiable images or data included in this article.

## Author contributions

YL and JL contributed to conception and design of the study. CW and JD organized the database. SZ and YW performed the statistical analysis. YY and YL wrote the first draft of the manuscript. JD wrote sections of the manuscript. All authors contributed to manuscript revision and read and approved the submitted version.
